# Reviewed article: Cordeiro DC, Aguiar EB, Luiz RR, Sacramento DS, Paolino PLW, Gonçalves MJF, Trajman A. Effect of successive tuberculin skin test readings on healthcare professionals’ proficiency: an operational observational study in five high-tuberculosis-burden Brazilian municipalities, 2023-2024. Epidemiol Serv Saude. 2025:34;e20240795

**DOI:** 10.1590/S2237-96222025v34e20240795.c

**Published:** 2025-10-31

**Authors:** Marilia Mastrocolla de Almeida Cardoso

**Affiliations:** 1 Ministério da Saúde, Brasília, DF, Brazil Brasília DF Brazil

## First round comments

Prezados autores, recomendo fortemente todas as observações apresentadas pelos revisores, reforçando a necessidade de detalhar o processo de realização das comparações uma vez que nem todos os potenciais leitores de um estudo como este conhecerão termos ou processos mencionados. Um exemplo de sentença: “No entanto, algumas estratégias complementares foram utilizadas para mitigar a dificuldade de alcançar o número de 100 braços com alguma reação palpável.”

Seguem abaixo observações complementares:

- Quem são os multiplicadores? O que fazem? Por que são considerados padrão-ouro?

- Este trecho não ficaria melhor no método? “Para a tomada de decisão clínica (prescrever ou não o tratamento preventivo da tuberculose), a sensibilidade e especificidade são mais importantes do que a concordância em torno de valores que não mudam o resultado de negativo para positivo e vice-versa. Desta forma, analisamos a sensibilidade e especificidade da leitura do capacitado utilizando os valores de corte de 5 mm e 10 mm - utilizados no Brasil (3), ...”

- Reforço a importância de rever o título e o objetivo, para que fiquem mais alinhados aos achados.

Recommendation: Major revision

## Second round comments

Prezados autores, 

O grande desafio deste estudo é o fato dele ser um detalhamento operacional muito específico. O que requer um entendimento prévio do leitor, ou então, um texto um pouco mais completo. Neste sentido, segue a proposta para mais alguns ajustes:

1) Introdução

- Inserir referência: “ No Brasil, uma das possíveis barreiras à ampliação do tratamento preventivo da tuberculose é o reduzido número de capacitadores e de profissionais de saúde certificados para realização da prova tuberculínica, exame fundamental na avaliação da infecção latente por tuberculose em grupos de risco.”

2) Método

- Descrever brevemente o que é o ExpandTPT (objetivo, executado por quem, desde quando etc)

- Substituir ou explicar a palavra “endurado” pois não é comumente utilizada como uma forma padrão.

- Explicar o que significa “censura na última leitura”.

3) Ilustrações

As ilustrações e seus títulos não atendem as instruções da RESS. Como se trata de segunda rodada sem que os ajustes tenham resolvido as pendências tomo a liberdade de enviar em anexo modelo que os autores devem considerar conforme pertinente. Ressalto que esta oportunidade de ajuste deve ser atentamente considerada pelos autores para evitar rejeição do manuscrito por inobservância das instruções, que deveria ter ocorrido antes da submissão.

Para conhecimento, as instruções estipulam “Mantenha a consistência ao alinhar dados, símbolos e texto. Crie cabeçalhos curtos, autoexplicativos e com unidade de medida, se aplicável. Todos os dados das células devem ter a mesma natureza do que foi informado no cabeçalho da tabela. Não é permitido, por exemplo, em uma tabela cujo cabeçalho informa contagens, incluir média e desvio padrão. Especifique as estatísticas relatadas nos cabeçalhos (ex.: “Média±DP”, “Mediana (IIQ)”, “n (%)”) e informe as unidades de medida nas colunas, quando aplicável, sem repetir as unidades em cada célula (ex.: %). Informe a unidade na linha da variável (ex.: “Renda (salários mínimos)”; “Faixa etária (anos)”) e remova repetições nas categorias da variável. Utilize hífen (“-”) para intervalos numéricos das categorias (ex.: 0-4) e assegure compatibilidade entre as categorizações apresentadas nas tabelas e aquelas informadas nos métodos, com consistência em todo o texto.”

A partir dos pontos indicados para a Tabela 1 e das instruções da RESS, favor ajustar a Tabela 2 e seu título. Além disso, falta separador de milhar nos numerais > 999 e as categorias deveriam ser identificadas como “0-4”, “≥10”, evitando a repetição da unidade (mm), por exemplo. Ressalta-se que esses pontos constam nas instruções.

Neste sentido, as cidades deveriam ficar nas linhas e as unidades nas colunas (modelo em anexo); o título deveria ser algo como “Tabela 1. Média, desvio padrão (DP), valores mínimo e máximo, mediana e intervalo interquartil (IQ) das leituras da prova tuberculínica segundo o município. Manaus, Porto Alegre, Recife, Rio de Janeiro e São Paulo, 2023-2024 (n=168)”

A Figura 1 precisa ser melhor refletida pelos autores. A unidade de medida não é clara. Seria a leitura em mm? O título informa que se trata de profissionais, multiplicadores e cidades. Há somente uma linha de tendência e não há identificação do que seria a linha horizontal no meio do gráfico (seria a média)? Após resolver essas questões de racional epidemiológico (atualmente não parece fazer sentido), é necessário seguir as instruções conforme texto abaixo. A escala do eixo Y precisa ser reduzida de modo ao conteúdo do gráfico ser melhor visualizado (há muitos espaços em branco desnecessariamente); o eixo poderia ter abrangência entre 0,5 e -0,5, por exemplo. Falta identificar o “n” (n=xx) no final do título ou em cada ponto de leitura, uma vez que pode ter variado o quantitativo em cada ponto. A partir destas questões, as figuras 2 e 3 precisam ser totalmente reestruturadas (ajustar a escala do eixo Y, explicar o que é a linha horizontal central). Não há necessidade, por exemplo, da figura 3 ser apresentada em mosaico; a especificidade e sensibilidade seriam linhas distintas no mesmo gráfico.

“Identifique cada eixo claramente com o nome da variável, as unidades em que a variável é plotada e quaisquer multiplicadores associados às unidades. Indique claramente o ponto zero dos eixos X e Y do gráfico, especialmente se um ou ambos os eixos não começarem em zero. Organize as escalas para que os valores do eixo Y aumentem de baixo para cima e os valores do eixo X da esquerda para a direita. Ajuste as escalas para maximizar o uso do campo de dados. Inclua apenas divisões e rótulos essenciais, lógicos e geralmente equidistantes nas escalas. Minimize as divisões desnecessárias e as marcas de escala sem rótulo.”

Recommendation: Minor revision

## Third round comments

Prezados autores, e

Essa será a última oportunidade de ajuste, caso permaneça sem ajustes suficientes, o processo será encerrado.

A Figura 2 ainda carece de ajustes, conforme indicamos abaixo:

- O título precisa refletir o conteúdo da figura. Consta que é o percentual. Os autores devem ajustar o título para informar o que são os números apresentados dentro da figura ou remover tais números que constam dentro da figura.

Caso opte por continuar informando os números que constam dentro da figura, é necessário ajustar para esses números ficarem exatamente acima do ponto da linha ao qual se referem. A conotação atual (ex.: n= 166 - 161) é confusa, por parecer uma subtração, e deve ser removida, bem como a nota sobre esse número no canto inferior direito da área do gráfico.

Outros pontos para aprimorar a clareza e comunicação dessa figura e que devem ser refletidos e adaptados para as demais:

- Remover “(diferenças até 2mm)”, pois é uma definição metodológica do que foi considerado como “concordância”, a unidade de medida/desfecho principal dessa figura.

- O termo concordância, por outro lado, usualmente é aplicado para cálculo de Kappa. Sugere-se que os autores reflitam e padronizem o texto adequado em todo texto, garantindo consistência nas ilustrações, títulos, texto, resumo etc.

- Ajustar a identificação do eixo y para “concordância [ou outro termo mais adequado] (%)” e remover a casa decimal “,0” das linhas do eixo.

- O intervalo do eixo y precisa ser ajustado. Na forma atual, de 0 a 100, não ilustra adequadamente, já que os dados variam entre >75 a 90%. O eixo precisa ser ajustado para intervalo menor, ex: 70 a 100, de modo a permitir visualização dos dados mais relevantes e ter menos espaços em branco na figura. Esse aspecto foi demandado na última rodada e não foi observado pelos autores. Demais figuras precisam ter ajuste semelhante, conforme pertinente.

Recommendation: Minor revision

